# COSMOS: Collaborative, Seamless and Adaptive Sentinel for the Internet of Things

**DOI:** 10.3390/s19071492

**Published:** 2019-03-27

**Authors:** Pantaleone Nespoli, David Useche Pelaez, Daniel Díaz López, Félix Gómez Mármol

**Affiliations:** 1 Department of Information & Communication Engineering, University of Murcia, Calle Campus Universitario, 30100 Murcia, Spain; felixgm@um.es; 2 Department of System Engineering, Colombian School of Engineering Julio Garavito, AK 45 (Autonorte), Bogotá 205-59, Colombia; david.useche@mail.escuelaing.edu.co (D.U.P.); daniel.diaz@escuelaing.edu.co (D.D.L.)

**Keywords:** Internet of Things, sentinel for the IoT, intrusion detection system, smart home, machine learning, malware detection, threat intelligence

## Abstract

The Internet of Things (IoT) became established during the last decade as an emerging technology with considerable potentialities and applicability. Its paradigm of *everything connected together* penetrated the real world, with smart devices located in several daily appliances. Such intelligent objects are able to communicate autonomously through already existing network infrastructures, thus generating a more concrete integration between real world and computer-based systems. On the downside, the great benefit carried by the IoT paradigm in our life brings simultaneously severe security issues, since the information exchanged among the objects frequently remains unprotected from malicious attackers. The paper at hand proposes COSMOS (Collaborative, Seamless and Adaptive Sentinel for the Internet of Things), a novel sentinel to protect smart environments from cyber threats. Our sentinel shields the IoT devices using multiple defensive rings, resulting in a more accurate and robust protection. Additionally, we discuss the current deployment of the sentinel on a commodity device (i.e., Raspberry Pi). Exhaustive experiments are conducted on the sentinel, demonstrating that it performs meticulously even in heavily stressing conditions. Each defensive layer is tested, reaching a remarkable performance, thus proving the applicability of COSMOS in a distributed and dynamic scenario such as IoT. With the aim of easing the enjoyment of the proposed sentinel, we further developed a friendly and ease-to-use COSMOS App, so that end-users can manage sentinel(s) directly using their own devices (e.g., smartphone).

## 1. Introduction

The Internet of Things (IoT) paradigm envisions a world where *everything* is connected together. That is, each everyday-life object will be reachable from the Internet with its own IP address through the already existing network infrastructures [1]. This innovative concept carries a profound revolution in how we interact with our daily environment, where massive amounts of information will be constantly flowing in our surroundings. The IoT devices may be located in home appliances, buildings, vehicles or monitoring infrastructures, and they might be remotely controlled by software services that allow them to be managed [2]. Under this paradigm, some vital domains will be enhanced, such as health-care [3], resulting in great economic and social benefits. The IoT potentialities soon attracted the attention of a vast audience, both from the academia and industry [4], exploding in a rapid growth which caused 6 billion devices connected in 2016 and foresees 21 billions of smart objects in 2020 [5]. From an economic viewpoint, Cisco Systems (San Jose, California, CA, USA) predicts a potential $14.4 trillion income as a result of the combination of increased revenues and lower costs for companies from 2013 to 2022 thanks to the adoption of IoT technologies within their businesses [6].

Despite the great benefits provided by the IoT integration within the real world, the enormous flow of information autonomously generated by the IoT devices becomes attractive for ill-motivated actors which try to manipulate and get unauthorized access to such data [7]. Recent research has proven that IoT devices can be remotely hacked by exploiting certain vulnerabilities, thus causing potential damage to final users [8,9]. Additionally, the intelligent devices have been severely compromised with the Mirai malware, which in late 2016 utilized the IoT nodes to create a botnet to launch massive DDoS (Distributed Denial of Service) attacks all over the globe [10]. In such scenario, security solutions ensuring protection for the IoT nodes definitely represent a primary need. To this extent, researchers worldwide have struggled to propose solutions to address the posed security challenges [11]. Unfortunately, due to the myriad of communication protocols, the resource-constrained nature of the IoT nodes, and the massive amount of information flowing, traditional security measures may not be directly enforceable (e.g., conventional cryptography, etc.), obliging to propose novel methodologies instead [12].

Furthermore, the IoT paradigm has been successfully integrated into house appliances, introducing the concept of *smart home*. In such scenarios, the overwhelming connectivity of the *things* will surely enhance our quality of life, since the IoT objects will be able to communicate autonomously to provide services to the house inhabitants as an ultimate goal [13]. As for its drawbacks, the privacy issues raised by such a continuous and transparent flow of information will become a cornerstone [14]. That is, most of the communications among the IoT devices are performed through wireless communications, which are inherently vulnerable to several attacks (e.g., eavesdropping, man in the middle, etc.) [15]. Moreover, one could argue that the common final users may not have the right expertise to keep their devices updated, plus the patches proposed by the vendors for these tiny devices are not so frequent.

To address the above-mentioned challenges, several solutions have been presented in the context of IoT security proposing, among others, lightweight protocols considering the resource-constrained nature of the IoT devices [16], or methodologies dealing with both the HW (hardware) and SW (software) layers of the IoT infrastructures [17]. Additionally, IoT intrusion detection architectures have been proposed in [3,18], where SIEM (Security Information and Event Management) technologies were responsible for managing the security events. Although these proposals look promising, very few consider the possibility of integrating multiple protection layers in a full-fledged solution to detect cyber incidents. The presence of different sequential layers allows one to exploit multiple defensive strategies, resulting in a more accurate protection. In addition, among the several proposals in the literature, there is a general lack of adaptability. Since IoT environments are characterized by the high dynamism and mobility of their nodes, we can claim that a solution capable of adapting to previously unknown threats is of vital importance. Additionally, we consider the collaborative skill crucial in the cyber threats detection task; that is, the entities in charge of detecting cyber attacks (i.e., the so-called sentinels) must be able to share autonomously information about the faced threats, so that other IoT entities may acquire the right expertise to eventually deal with the same circumstance in a timely fashion [19]. Finally, one could argue that a suitable IoT Sentinel must be portable and easy to deploy. That is, the final user must be able to relocate the sentinel effortlessly, requiring minimum configuration at any place where security must be provided.

In this paper, we present COSMOS (Collaborative, Seamless and Adaptive Sentinel for the Internet of Things), a novel approach to protect IoT nodes against cyber threats. The proposed solution is applicable, but not limited, to a smart home scenario. As an extension of the proposal in [20], COSMOS is able to seamlessly monitor and analyze the network traffic of the surrounding area, inspecting the captured frames to look for potential anomalies. Additionally, the generated events are centrally reported to a SIEM server in case of intrusions. Using a SIEM platform, the collected events are correlated to distinguish between malicious activities and false alarms. This in turn would increase the situational awareness, helping in identifying the optimal countermeasures to react to the cyber incidents [21]. In addition to the work proposed in [20], COSMOS leverages the combined capabilities of two IDSs (Intrusion Detection Systems) (i.e., Kismet and Snort) to perform an in-depth inspection of both the Ethernet and wireless frames flowing around the IoT Sentinel, checking for potential intrusions at network level. Furthermore, an Android application has been developed to remotely control the IoT Sentinel, thus demonstrating the capabilities of the proposed framework in a real use-case scenario for the final users. Specifically, the user can extensively manage the incoming events from COSMOS through real-time interactions, thus protecting the surrounding environment by getting informed of the ongoing security events in a timely fashion.

**Figure 1:**
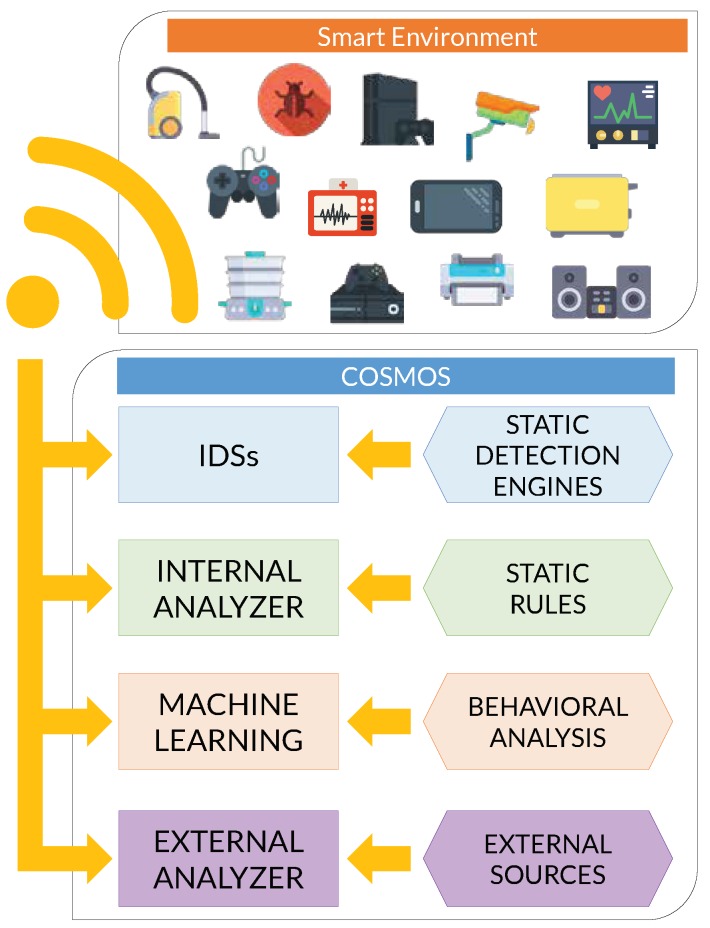
Abstract view of COSMOS.

The main contributions of this paper are summarized as follows:

- The proposal of COSMOS (see Figure 1) based on a defense-in-depth approach oriented to perform the main security functions for asset protection (identify, protect, detect, react and recover) within an IoT ecosystem.
- The design of a mechanism for sharing threat intelligence between IoT Sentinels, which is critical to guarantee collaboration, reduce incidents’ response time and prevent attacks.
- The conception of an adaptive mechanism that allows for updating defense capabilities in IoT Sentinels, making them resilient to an evolutionary threat environment.
- The development and evaluation of COSMOS as a full-fledged security framework aiming to be as much seamless as possible to users, automatic, portable, easy to deploy and manageable in a pragmatic way through a mobile application.

The remainder of this paper is structured as follows: In Section 2, the COSMOS solution is presented together with its protection-oriented security goals, as well as an implementation to prove its feasibility. Next, Section 3 shows the principal use cases where COSMOS can operate. Then, in Section 4, we demonstrate through exhaustive experiments the accuracy and suitability of COSMOS to promptly and efficiently detect cyber threats, adding an in-depth discussion on the obtained outcomes. In Section 5, the developed Android application is presented, showing its capabilities. Section 6 analyzes the most prominent works proposed in the field, highlighting their pros and cons and adding a comparative overview. Last but not least, Section 7 concludes the work, summarizing the main outcomes and identifying potential future research directions.

## 2. Collaborative, Seamless and Adaptive IoT Sentinel

This section describes the main aspects of the COSMOS solution, covering the definition of security goals and the explanation of its key components. COSMOS monitors all the traffic traversing around its nearby environment (e.g., a smart home scenario or a smart office) and analyses it using, sequentially, each of its defense rings, looking for potential cyber incidents.

In order to concretely shield the surrounding IoT devices, the proposal presented in this paper aims to meet the next security goals:

1. **Offer essential security services**: COSMOS should provide a set of essential security services for IoT devices, i.e., identify those IoT devices to be protected, deploy security countermeasures to protect them, detect attack attempts, react automatically to incidents and recover the normal operation of the IoT ecosystem.
2. **Share threat intelligence**: Design a collaboration mechanism between IoT sentinels allowing for sharing threat intelligence information which can be useful in the treatment of security incidents and help to prevent IoT attacks.
3. **Adapt to the threat environment**: Develop a mechanism to make COSMOS adaptive within continuously evolving threat environments, making it resilient against threats that have been detected in the past by another IoT Sentinel [22].
4. **Follow a psychological acceptability approach**: The implementation of the proposal should provide transparency to the users of the framework, automation of operation, portability, easiness of deployment and management in a pragmatic way.

One could easily notice that the combined action of the above-mentioned goals can result in an optimal protection level for the *intelligent* devices, while requiring minimum interaction with the final user.

**Figure 2:**
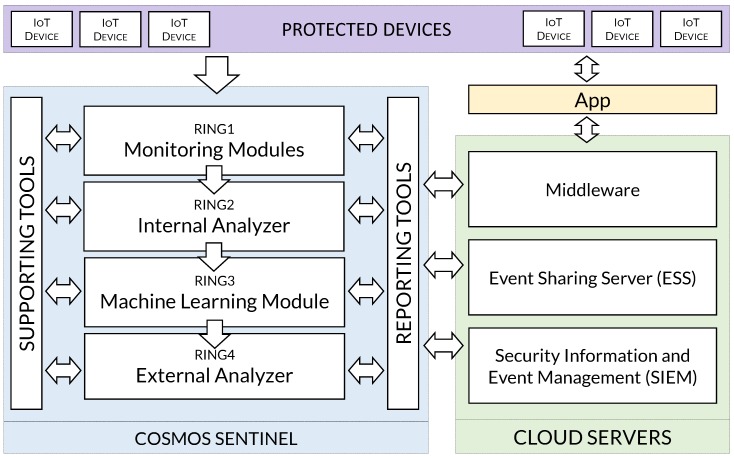
High level architecture of COSMOS.

The architecture of the IoT Sentinel is composed of different modules as depicted in Figure 2. Some of the modules are lightweight and the computing power to execute them is limited, so they can run locally in the IoT Sentinel. On the other side, other modules definitely must be executed remotely, e.g., in the cloud, as they require extensive resources.

Despite the important security functions performed by all the modules, those developing the most critical tasks in the IoT Sentinel correspond to the Monitoring Modules, the Internal Analyzer, the Machine Learning Module and the External Analyzer, as we will see later. These modules constitute the security shields which are the core of the IoT Sentinel, so if a suspicious file or sample goes through one ring (e.g., a document or an executable browsed by one user within the protected scenario), it is evaluated by the next ring, following a defense-in-depth philosophy. In the first layer, the samples are intercepted by the monitors (i.e., Monitoring Modules), which in turn are capable of analyzing them using powerful IDS rule-based engines.

The second protection ring (i.e., Internal Analyzer) uses static rules to check whether a captured sample (an App or executable downloaded by one of the protected IoT devices, for instance) is malware. The third safeguard (i.e., Machine Learning Module) performs a classification labor over the behavior of the sample. Last but not least, the final shield (i.e., External Analyzer) is in charge of sending the sample to an external service to retrieve a malware report. The IoT Sentinel also contains additional modules that perform complementary security functions like: vulnerability scanning, incident information sharing and correlation of events. Moreover, due to the above-mentioned performance reasons, other components are deployed in the cloud, such as: SIEM and the event sharing server.

For the sake of clarity, the different modules composing the COSMOS architecture are clustered into different categories depending on their functions, namely: monitoring, analyzers, supporting tools and cloud servers, as reported in the following sections. In order to test the feasibility of our proposal, each of those components has been implemented resorting to open-source and widely employed tools, leveraging their strong supporting community and interoperability.

### 2.1. Monitoring Modules

These modules monitor the network traffic and surrounding IoT devices looking for potential threats and existing vulnerabilities. Moreover, they are also responsible for feeding both (i) the Analyzers modules with potentially suspicious files or samples that must be evaluated, and (ii) the SIEM Client module (within the reporting tools) with security events that must be correlated and analyzed to determine the existence of an attack. More specifically, the modules belonging to this category are the *Ethernet monitor*, the *WiFi monitor*, and the *Vulnerability scanner*.

First, the Ethernet monitor audits and detects intrusions passing through the Ethernet channel of the IoT devices. The implementation should be able to sniff the network traffic and inspect files being passed between IoT devices and Internet servers, so that these can be checked by the Analyzer modules. In addition, the Ethernet monitor sends the alerts generated by the matched IDS rules to a SIEM server that correlates the reported security events with other information received by other modules, looking for security incidents as an ultimate goal. The tool used by COSMOS to monitor the Ethernet traffic is Snort [23], a free and open-source network threat detection engine. The Snort engine is capable of performing real-time intrusion detection, inline intrusion prevention, network security monitoring and offline PCAP (packet capture) processing [24].

Next, the WiFi monitor checks and detects intrusions passing through the IEEE 802.11 channel of the IoT devices. Also in this case, this module should be able to sniff the network traffic of the surrounding wireless area aiming at finding possible malicious frames. If any suspicious packet flowing among the IoT devices is detected, it is passed to the Analyzer modules for additional investigation. Similarly to the Ethernet monitor, the WiFi monitor is also able to send the alerts generated by the matched wireless IDS rules to the SIEM server, thus adding meaningful information on the possible intrusions (e.g., multi-step attacks). The implementation used to monitor the network traffic in the IEEE 802.11 standard is Kismet [25], a network detector, passive sniffer and IDS. It works with any wireless card supporting raw monitoring mode. By sniffing the raw packets on the wireless channels, it is able to infer on the features of the observed network, clients, cryptographic settings, and so on.

In addition to the monitors, the Vulnerability scanner searches on the network for vulnerable IoT devices and sends the gathered information to the SIEM server, who correlates such alerts with security events reported by other modules (e.g., Ethernet and WiFi monitors). The implementation used to scan for vulnerabilities is OpenVAS [26], a well-known framework composed of several services and tools offering a vulnerability scanning and vulnerability management solution [27].

### 2.2. Analyzer Modules

These modules are in charge of analyzing certain files (e.g., installation files) traversing the IoT network as well as URLs queried by the IoT devices in order to determine whether the sample is malicious (i.e., malware). These modules are invoked by the Evaluator module (as reported in Section 2.3), once one of the aforementioned monitoring modules has captured a file or sample. In particular, COSMOS has been equipped with an *Internal Analyzer*, a *Machine Learning Module*, and an *External Analyzer*.

Concretely, the Internal Analyzer is responsible for (i) receiving the suspicious files or samples from Evaluator module, (ii) analyzing them locally using static analysis and (iii) returning the results to the Evaluator module, indicating whether the sample was considered as malware or not. The Internal Analyzer uses Yara [28] rules, a project aimed at helping malware research to identify and classify malware samples [29]. This is done by creating descriptions of the malware families based on textual or binary patterns of the sample. Yara may be combined with other modules to enforce the rules, thus achieving better results. If any of the enabled rules matches, the Internal Analyzer returns which Yara rules were activated.

In addition, the Machine Learning Module (ML) is in charge of classifying an unknown received sample as goodware or malware, and returning the classification result to the Evaluator module. First, the ML module queries the Reversor module (see Section 2.3) for a report, which contains the set of permissions requested by the sample. Such report is then passed to the ML model in order to obtain the actual classification of the sample. The ML model is developed using Weka [30], a toolbox that offers a variety of models for data mining and machine learning [31]. In the context of this article, the classification model implemented uses the Random Forest algorithm, as it generally converges rapidly toward optimum results. The machine learning model was trained using 100 goodware and 100 malware samples collected from publicly available repositories, such as Koodous [32], apkmirror [33] and Drebin dataset [34]. In particular, the proposed model was tested using 20-fold cross-validation.

Finally, the External Analyzer receives suspicious files or samples from the Evaluator module and sends them to an external server which analyzes them and determines whether they are malicious or not. The answer retrieved from that external server is returned back to the Evaluator module. The External Analyzer uses VirusTotal [35], a free online service that analyzes files to identify viruses, worms, trojans and other kinds of malicious content detected by antivirus engines and website scanners [36].

### 2.3. Supporting Tools

These modules conduct different actions invoked by the other modules within the IoT Sentinel, thus helping the overall working cycle of the COSMOS framework. In the following, the Reversor and Evaluator modules are detailed, explaining their functionalities and concrete implementation.

Firstly, the Reversor module performs reverse engineering over a file, App or executable, retrieving hashes, entry points, libraries, permissions and any information that may help to identify a sample as malicious. In the context of this paper, the Reversor module is implemented using a combination of functions provided by Radare2 [37] and Androguard [38]. Radare2 (r2) is a reverse-engineering tool with forensic features [39]. In turn, Androguard is a framework developed in Python allowing to interact directly with the code of an application and to perform forensic functions over a suspicious Android sample. The Reversor may be invoked by the Internal Analyzer, the Machine Learning Module and the Reporter, respectively.

In turn, the Evaluator module may be mentioned as one of the most important modules of the COSMOS framework, since it acts as an orchestrator. Specifically, it gets a suspicious file or sample collected by the Monitoring modules and sends it to the Internal Analyzer. If the Internal Analyzer does not identify the sample as malware, subsequently the Evaluator module sends the sample to the ML module for evaluation. If also in this case the sample is not recognized as malware, the Evaluator module finally sends it to the External Analyzer. If, and only if, a sample is not detected as malware by any of the above-mentioned layers, then it is considered as goodware. This conservative approach helps the entire framework to achieve better results in terms of correct detection, as discussed in Section 4. the sample is declared as malware by means of a correct detection, the Evaluator module invokes the Alert Generator and Reporter modules (see Section 2.4) in order to inform other IoT Sentinels about the detected threat. The implementation of the Evaluator component is an ad hoc module implemented in Python.

### 2.4. Reporting Tools

These modules are in charge of generating appropriate reports of the cyber incidents detected, as well as of connecting the IoT Sentinel with the cloud services it consumes. Here, we find the SIEM Client, the Event Sharing Client, the Reporter module and the Alert Generator module, as detailed in the following.

The *SIEM Client* connects the IoT Sentinel with the SIEM and might be invoked by the Monitoring modules. This component has been developed as a self-made Java module.

Then, the *Event Sharing Client* is responsible for the communication between COSMOS and the Event Sharing Server located in the cloud. The implementation has been done through a client that uses the Malware Information Sharing Platform (MISP) Python API, so the IoT Sentinel can send events toward an Event Sharing Server. Specifically, MISP is a framework that allows sharing information regarding detected threats with other IoT Sentinels. The Event Sharing Server is implemented using a MISP instance, as we will describe in Section 2.5.

In turn, the Reporter module is capable of generating a report using the information gathered from the Reversor and sending it to the Event Sharing Server for sharing purposes and further analysis. In case that the Internal Analyzer is not able to classify a sample as malware, but either the ML module or the External Analyzer achieved such task, the Reporter module is also able to generate a static Yara rule using the hash of the sample calculated by the Reversor. By doing so, the new generated rule can be retrofitted to the Internal Analyzer, thus avoiding future incorrect detections and adapting to the previous unknown threats. The Reporter module is implemented in Python and is invoked by the Evaluator when any of the four rings detects a sample as malware.

Last, but not least, the Alert Generator creates an alert to warn the final users about the malicious file that has been detected and about the actions that were taken (either the elimination of the file or patching the devices). The implementation used is a Python module that employs a mail API to send an email to a predefined address. This email indicates which tool has performed the detection of the file and which action was taken.

### 2.5. Cloud Servers

These components represent the modules on the cloud side and contain services that the IoT Sentinel consumes during its working cycle, as illustrated in Figure 2.

Firstly, the SIEM Server behaves as a rule correlation engine. It is also able to give instructions to the IoT Sentinels, so that they can react when an attack is detected. The instructions may be a collection of scripts with OS (Operative System) commands, one for each of a variety of situations. The tool used in the context of this paper is OSSIM [40], a SIEM with event collection, normalization and correlation. It is capable of performing asset discovery, vulnerability assessment, intrusion detection and behavioral monitoring [41].

Furthermore, the Event Sharing Server (ESS) receives events from the Event Sharing Client and shares them with other IoT Sentinels so that they can generate appropriate countermeasures to prevent that a specific malware infects further IoT devices (e.g. create a Yara rule). The implementation of the Event Sharing Server is done through MISP which is an open-source software that gathers, shares, stores and correlates indicators of compromise (IoC) regarding a variety of security incidents [42]. Indicators of compromise and malware information are clustered in a MISP event, which is the information unit that is actually shared between MISP instances. By doing so, the objective of being collaborative, as already described, can be achieved by leveraging the capabilities of MISP.

Finally, the Middleware acts as a service provider for both the IoT Sentinel and the App, as we will see later in Section 5. Specifically, the Middleware is in charge of managing the communication between the entities by providing a queuing service. The implementation has been done through an ad hoc Python module.

## 3. IoT Sentinel Use Cases

The interactions among the elements presented in Section 2 result in an effective protection level to the surrounding IoT devices. For the sake of clarity, the behavior of the Sentinel is detailed in the following five cases, based on which detection layer is able to unveil the malicious file.

### 3.1. Case 1: Suspicious Frame Detected by the Monitoring Modules

In this case, a frame is intercepted by the Monitoring modules, which, using the IDSs’ detection engines, are able to correctly identify as potentially malicious. Consequently, the generated event is reported to the the IMS Client, in charge of sending it to the cloud SIEM Server. Such event is then stored and correlated with other security events, helping in distinguishing between false positives and actual cyber-attacks. This in turn will increase the overall situational awareness for the final users, which can smoothly choose which reaction shall be fired.

### 3.2. Case 2: Suspicious File Classified as Malware by the Internal Analyzer

Here, the suspicious file is captured by the Monitoring modules and the Evaluator module sends it to the Internal Analyzer. This layer evaluates the suspicious sample using static Yara rules. As part of the execution of these rules, the Reversor is invoked to obtain forensic sample information. If at least one of the Yara rules matches with the sample, the latter is classified as malware and the Evaluator is informed. Then, the Evaluator generates an alert and a report using the Reporter module. Specifically, the Reporter module checks with the Reversor to get the features regarding the malicious file that are required to build a MISP event. The generated MISP event is sent by the Reporter to the Event Sharing Client who in turn sends it to the Event ESS, i.e., MISP server. The sample itself is also sent to the ESS as an attribute of the created MISP event.

### 3.3. Case 3: Suspicious File Classified as Malware by the Machine Learning Module

In this case, the suspicious file is intercepted by the Monitoring modules and the Internal Analyzer classifies the sample as goodware. Consequently, the Evaluator sends the sample to the Machine Learning module which generates a report of the sample using the Reversor, specifically Radare 2 or Androguard, to obtain interesting information about such sample. The report is then sent to a previously trained machine learning model to classify the sample as goodware or malware using a Random Forest classificator that is executed using Weka libraries. If the sample is classified as malware, the Evaluator is informed and it generates an alert and a report using the Reporter module. The Reporter module asks the Reversor in order to get the features regarding the malicious file that are required to build a MISP event. Such MISP event is sent by the Reporter to the Event Sharing Client who is in charge of, in turn, sending it to the ESS, i.e., MISP server. The sample itself is also sent to the ESS as an attribute of the created MISP event. Finally, the Internal Analyzer autonomously generates an ad hoc rule which will detect the malicious file in the future.

### 3.4. Case 4: Suspicious File Classified as Malware by the External Analyzer

In this case, the suspicious file is captured by the Monitoring modules and neither the Internal Analyzer nor the ML Module are able to detect the sample as malware. Thus, the Evaluator sends the sample to the last layer (External Analyzer) which consumes the services of VirusTotal. The result of the analysis made by VirusTotal is a sample report identifying it either as malicious (by one or more detectors included in VirusTotal) or harmless. If the sample is classified as malware, the Evaluator is informed and it generates an alert and a report using the Reporter module. The Reporter module invokes the Reversor in order to get the features regarding the malicious file that are required to build a MISP event. Such MISP event is sent by the Reporter to the Event Sharing Client who also sends it to the ESS, i.e., MISP server. The sample itself is sent as well to the ESS as an attribute of the created MISP event. Finally, the Internal Analyzer autonomously generates an ad hoc rule which will detect the malicious file in the future.

### 3.5. Case 5: Suspicious File Classified as Goodware by the Detection Layers

Here, the suspicious file is captured by the Monitoring modules and the protection rings sequentially classify it as non-malicious. In this case, the suspicious file is assumed to be goodware, following a conservative approach. However, applying behavioral dynamic analysis (future work), such file could be subsequently unveiled in case it actually performs like a malware [43].

## 4. Experiments

Several experiments were conducted on the proposed architecture to prove its detection capabilities. COSMOS was deployed on a Raspberry Pi 3 model B, equipped with a quad core 1.2 GHz CPU, 1 GB RAM and Raspbian OS, which is optimized for its operations. We decided to test the IDS and malware detection capabilities separately, since one could say that such operation modes impact the performance in a different way, and it may be interesting studying them solely. Thus, the results of the experiments on the COSMOS IDSs performance are presented in Section 4.1, while the malware detection capabilities of the proposed architecture are discussed in Section 4.2.

### 4.1. Testing COSMOS Capabilities as an Intrusion Detector

This section outlines the experiments performed to assess the capabilities of the COSMOS architecture as the host of the described IDSs (i.e., Snort and Kismet). For the ease of readers’ understanding, the experiments settings are reported in Section 4.1.1, while a significant analysis of the results is carried out in Section 4.1.2. For additional details, readers are referred to [44].

#### 4.1.1. Settings

In order to argue on the performance of the COSMOS IDSs, we reproduced a scenario in which an external attacker performed some malicious activities against typical devices belonging to a home network scenario, i.e., a wireless router giving connectivity to a smartphone. In particular, the setup of the testing environment is reported in the following:

- Raspberry Pi 3 Model B uses the Raspbian OS with the Linux kernel version 4.1.19, Snort version 2.9.8.0 and Kismet version 2016-01-R1. The Sentinel performed the detection duties using two wireless interfaces: the Pi built-in WLAN chip communicated with OSSIM through Rsyslog, and an external Alfa AWUS036H USB antenna sniffed wireless traffic in the surrounding area.
- Laptop running a Kali 2.0 distribution, used for penetration testing [45], with Linux kernel version 4.3.0. It acts as the attacker in our scenario.
- Smartphone Galaxy Nexus I9250 running Android version 4.3, acting as the victim of the attacks performed by the attacker.
- Linksys Wireless-G Broadband Router (WRT54GS v6) with a twofold responsibility: on the one side, it gave connectivity to the testing sub-network with an intentionally weak WEP (Wired Equivalent Privacy) encryption. On the other side, it became also a victim of the wireless attacks performed by the attacker to gain the encryption key.
- Desktop PC running OSSIM version 5.2.2 with All-in-One installation.

The performance evaluation was conducted in a shared environment, where multiple devices communicated among them using several access points. Thus, the monitor interface captured the traffic from different sub-networks. For those frames, Snort was not able to start the detection process, since their payload resulted to be encrypted. Kismet, instead, inspected also this traffic as its rules are focused on the devices’ *behavior* and on possible 802.11 header anomalies. On the contrary, attacks performed in our sub-network were correctly detected by COSMOS, as malicious packets were decrypted by Kismet and dispatched to Snort, which in this case is able to directly analyze them. It has to be noted that the two tested IDSs were running on separate CPUs in order to avoid conflicts due to possible context switches. Specifically, CPU0 was hosting Kismet, while Snort was running on CPU1.

The tests have been performed for consecutive days, using an 8-hour time window (from 10 a.m. to 6 p.m.), in which we registered the highest amount of frames. By doing so, the Sentinel tried to capture wireless traffic with similar peculiarities. Within the above-mentioned window, the attacker performed random attacks against the victims with a fixed frequency (i.e., every hour). Simultaneously, a Python script was running on COSMOS to monitor the overall impact introduced by the IDSs and the log forwarding process on the system resources (i.e., CPU and RAM) and the number of intercepted packets, as reported in Table 1. In particular, Snort was tested with different configurations to argue on their resource consumption; that is, different rule sets (i.e., *connectivity*, *balanced* and *security*) and detection algorithms (i.e., *lowmem*, *ac-bnfa* and *ac-split*) were examined during the experiments, as summarized in Table 1. The latter were selected because they present the best overall performance according to Snort developers. On the other hand, Kismet was tested with the complete rule set enabled, limiting the maximum number of logged clients to avoid an excessive consumption of RAM.

#### 4.1.2. Results Analysis

**Figure 3:**
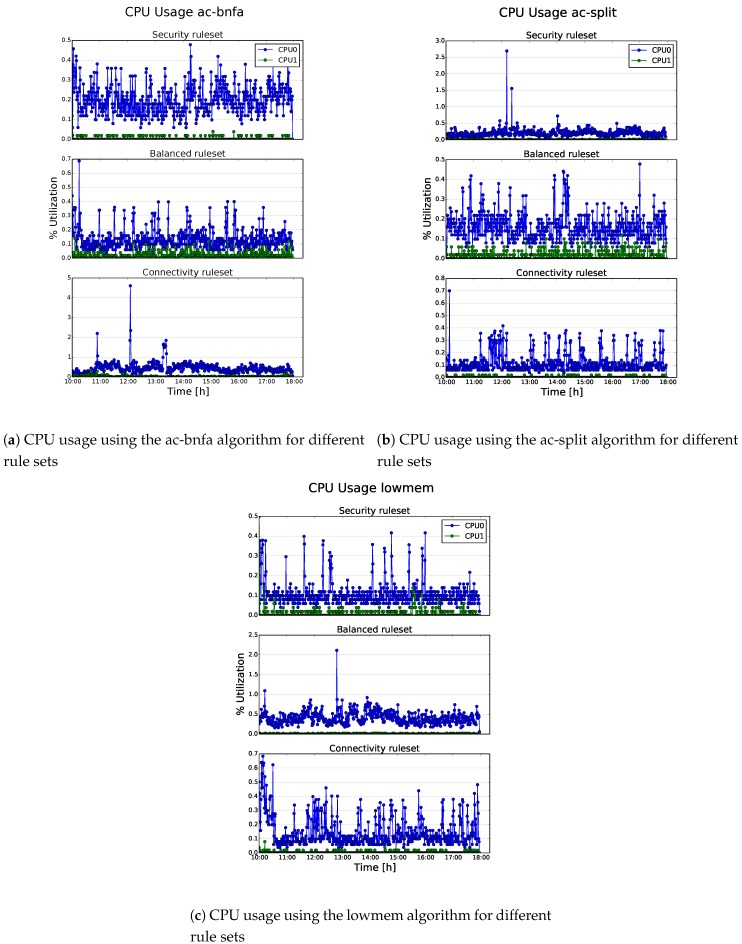
COSMOS IDSs CPU usage.

Figure 3a–c show the relationships between the CPU consumption of COSMOS and Snort’s tested detection engines. In particular, each figure analyzes also the impact of different rule sets together with the influence of the detection algorithm. The most interesting result showed in these graphs is that COSMOS is capable of handling gracefully the incoming stream of wireless traffic. Except for some outliers, the CPU usage stuck under 1% of utilization.

Regarding the different combinations of detection engines and rule sets, we can safely conclude that, in our experiments, the CPU consumption is not deeply influenced by the choice of one of them. In the graphs, the CPU1 usage (hosting Snort) is very low, and this behavior prevents us from arguing about possible impact differences of these combinations. On the other hand, the CPU0 usage (hosting Kismet) is slightly higher, since it was responsible for managing the interruptions generated by the packets’ capture. However, one could argue that the CPUs are not critically used during this experimental phase, since they need a much higher data rate to be stressed, as reported in [18].

**Figure 4:**
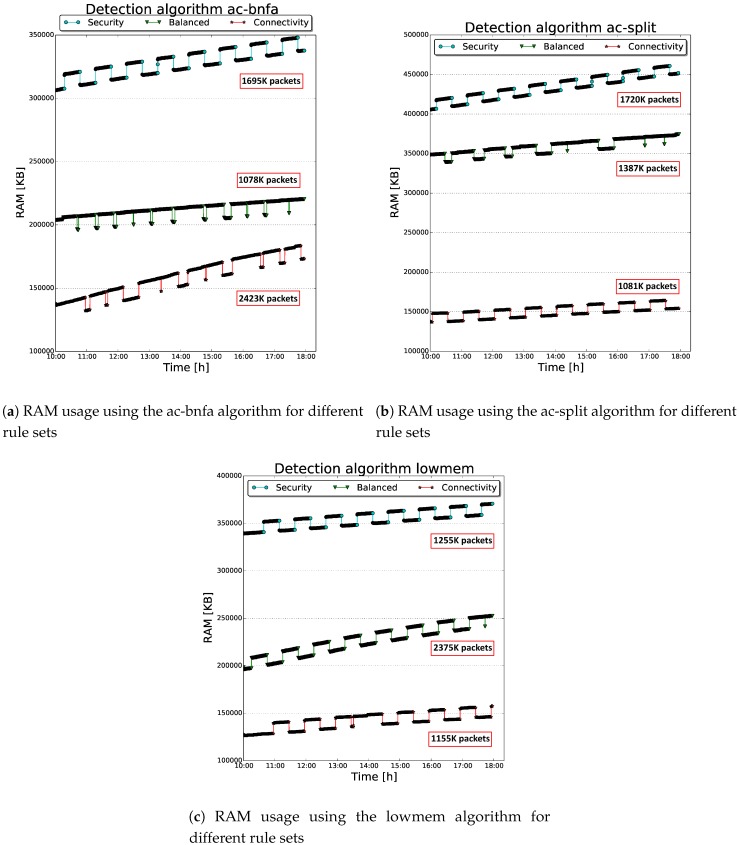
COSMOS IDSs RAM usage.

Additionally, Figure 4a–c show the relationships between the RAM consumption of the Sentinel and Snort’s tested detection engines. Specifically, for each analyzed engine, all the rule sets combinations are reported. There is a clear difference in RAM consumption between the three rule sets, with the Connectivity rule set that results to be the lightest and the Security rule set the most resource demanding of the three. Concretely, these plots clarify that, switching to a bigger rule set, results in higher RAM usage. The underlying reason is that the Snort’s detection engine, to perform its detection duties, loads into RAM all the rules belonging to the selected rule set. Therefore, a direct consequence is that the detection engine needs more memory to load the Security rule set (with its ~15 K rules) than the lighter Connectivity one. To this extent, the impact of Kismet’s ruleset on the RAM consumption is quite meager, since it contains 30 rules.

Although this behavior was expected, it is interesting to argue that also the choice of the detection algorithm has an impact on the RAM usage of our architecture. In particular, the *lowmem* and *ac-bnfa* engines seem to be more efficient than the *ac-split* engine. This is most likely due to the different internal data representation used by the detection algorithms. In particular, the ac-bnfa algorithm uses a Non-Deterministic Finite Automaton (NFA) [46], a more compact representation compared to the Deterministic Finite Automaton (DFA), used by the ac-split algorithm. In fact, any DFA is also an NFA, and the former can be obtained from the latter with a conversion operation. The resulting DFA will have exponentially more states than the original NFA. Therefore, the higher number of states that its automaton has to create causes the additional memory used by ac-split. In addition, the lowmem algorithm uses a tree data structure. Moreover, in this case the difference in memory consumption compared with the ac-split algorithm might be caused by the DFA used by the latter. This automaton results to be much more expensive in our experiments, in terms of memory usage, than a “simple” tree data structure used by the lowmem algorithm.

Furthermore, these plots highlight the inherent behavior of the proposed IDS architecture, emphasizing two salient aspects. The first one is represented by the curves’ shape; that is, the depicted RAM consumption shows a trend presenting periodical spikes. These peaks were mainly generated by the Snort detection process, which was evaluating the simulated attacks launched by the attacker in our sub-network. Recall that the attacker’s aim was to send random malicious frames against the victims (i.e., smartphone and wireless access point) at fixed time interval within the time window. As the attack started, Snort detected the intrusion and therefore it analyzed the potential malevolent frames so that the RAM usage increased for a certain time interval, causing consequently the above-mentioned spikes. These peaks result to be in each scenario ~10 MB, which is an acceptable amount considering the memory used by the different rule sets: in the worst case, it represents less than 10% of the total memory usage. Then, as the detection process ends, the occupied memory is freed.

The second salient feature depicted in Figure 4a–c is the increasing trend of the RAM usage plots. That is, during the 8-h time window, the combined actions of the IDSs dissipate the memory of the hosting system (i.e., the Raspberry Pi), showing a linear increasing trend. This behavior is mainly due to the Kismet’s internal mechanisms of *devices tracking*: during the detection process, special data structures are created by the wireless IDS to store relevant information about the active devices in the surrounding area. Specifically, when a device presents a behavior which could be marked as *suspicious* by the IDS, its characteristics are stored in RAM, so that it remains under observation. The number of devices followed by Kismet is controlled by the *maxbacklog* parameter. That is, the higher value is assigned to this parameter, the higher number of packets (and clients) will be stored and tracked. The backlog queue should be freed after a certain time interval, so that the RAM could be restored to the previous state. Nevertheless, some captured packets and consequently some structures are never deleted from the queue, resulting in an data accumulation. A direct consequence of this process is that the higher is the number of captured packets (more devices communicate), the higher is the slope (more RAM is used), as the plots clearly show. As experiments illustrate, Kismet operations must be reviewed in the future to guarantee a stable long-term operation.

The aforementioned experimental results in the IDSs capabilities of COSMOS demonstrate that, even in a heavily loaded environment, the proposed solution is able to handle and analyze the traffic efficiently. That is, we observed that the CPU usage never exceeded 5%, and the RAM usage never reached 500 MB utilization during the 8-hour experimental window. In addition, the attacks performed were indeed detected by the wireless IDS with high accuracy, and reported to the remote OSSIM server in a timely fashion. Thus, we can safely conclude that the proposed solution is suitable to protect IoT devices from potential cyber-attacks (i.e., intrusion attempts) aiming at exploiting the IoT network.

### 4.2. Testing COSMOS Capabilities as Malware Detector

This section outlines the experiments executed to determine the performance of the COSMOS framework as a malware detector. As previously described in Section 2, the malware samples are sequentially analyzed by three defensive layers (i.e., Internal Analyzer, ML Module, and External Analyzer), following the defense-in-depth approach. In particular, we decided to report separately the analysis of Android malware and other platforms (e.g., Windows, Linux, etc.), since they can be considered as two different use cases, thus implying different executions.

It is worth noticing that the current implementation of the ML layer is effectively working only for the Android malware detection, since the learning model is trained to distinguish such samples. Nonetheless, the operation flow is easily extensible to the detection of other malware extensions (e.g., .exe, .sh, .deb, etc.) by training another learning model on such files. Indeed, we are currently working on this extension which represents an interesting and challenging future work.

#### 4.2.1. Android Malware Detection

In this section, we describe the experiments conducted on the COSMOS architecture regarding the Android malware detection. Such experiments were carried out over a set of 5000 Android malware samples belonging to different families [34]. The samples were directly injected within the catching directory of the IoT sentinel so that the main detection module was fired against the incoming threats. To better argue on COSMOS performance, we decided to vary the sizes of the samples according to the division in [34]. Specifically, the selected size classes were: (i) less than 100 KB, (ii) from 100 KB to 500 KB, (iii) from 500KB to 1 MB, (iv) from 1 MB to 5 MB, and (v) more than 5 MB.

Regarding the machine learning model, the module was trained using a small subset of malware (i.e., 100 samples), and tested using 20-fold cross-validation. For the static detection layer, a set of 72 Yara rules was used instead. These rules were originally taken from the official Yara repositories concerning the mobile detection. To assess the sentinel’s behavior, the following parameters were measured, as reported in Table 2: (i) the overall *resource consumption* of the framework’s components (i.e., CPU and RAM consumption), (ii) *response time*, that is, the time a ring requires to give a response about a given sample, and (iii) *detection rate*, meaning the percentage of malware samples detected for a given experiment in each ring. We believe that the above-mentioned parameters are particularly critical in the context of IoT threats’ detection.

**Figure 5:**
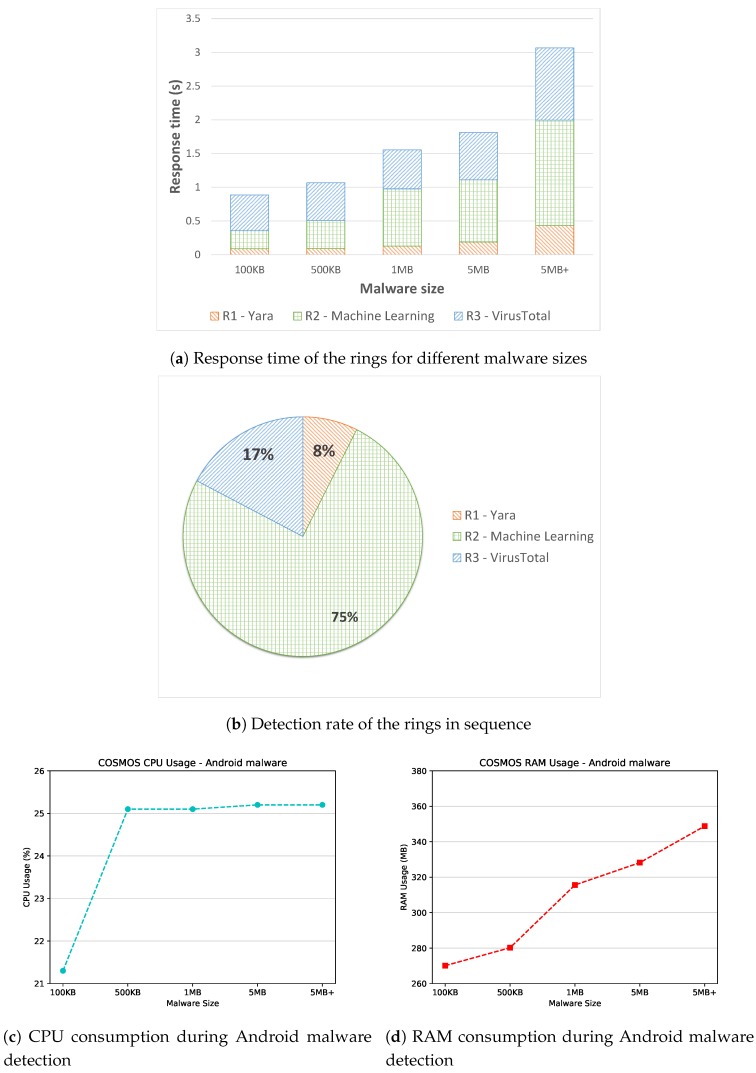
Results of the experiments conducted on COSMOS w.r.t Android malware detection.

Relevant results are shown in Figure 5, where the response time of the Sentinel for different malware sizes is depicted in Figure 5a, the detection rate of the three malware detection rings is showed in Figure 5b, and finally the resource consumption in terms of CPU and RAM is illustrated in Figure 5c,d, respectively.

Regarding the response time, Figure 5a illustrates the time required for the malware samples analysis of the three defense rings of COSMOS. It has to be stated that the response time has been calculated separately for the rings during this experiment to give a more accurate measure for each ring. Additionally, we decided to plot the median value of the collected time values, since this statistical measure is more robust to the possible presence of outliers in the results. As expected, the first ring (i.e., Yara) is the fastest within the architecture. This characteristic is mainly due to the fact that the Yara rules used to detect the peculiarities of the Android malware are relatively low in number (i.e., 72 rules). Moreover, since the enabled rules are signature-based, the time required by the detection engine to analyze them is negligible. Concerning the other rings, for malware sizes less than 500 KB, the machine learning module performs faster than VirusTotal. Later, this trend is inverted considering malware sizes greater than 500 KB. This outcome is justifiable looking at the different operations performed internally by the second and third ring. In fact, while the machine learning module checks the permissions required by the Android application, VirusTotal computes only the hash of the files and sends it to external databases through the Internet connection. Thus, while the response time of VirusTotal remains quite constant (except for very large malware samples, for which the time needed for the hash calculation becomes considerable), the time required by the machine learning tool increases linearly.

Another interesting result obtained during the time analysis experiments is represented by the trend of the response time of the different rings. That is, Figure 5a shows that the malware analysis time of the rings increases when the malware size grows. Specifically, the first ring (i.e., Yara) performs a static analysis of the malware code in order to detect possible matches with the enabled rules. Thus, the time required for this static analysis increases for bigger malware. Similarly, the second ring (i.e., the machine learning module) checks the permissions requested from the Android application during its execution in order to discriminate between goodware and malware. Therefore, the time demanded for the permissions analysis is dependent on the malware size, mainly for the time required for the decompilation and the permissions extraction. Likewise, as previously stated, the third ring (i.e., VirusTotal) needs to compute the hash of the input file and then send it online to external databases. Consequently, a bigger input file could imply a bigger hashing generation time before the hash can be actually sent to the VirusTotal API to obtain a malware report. On the other hand, when a sample hash is not found in the VirusTotal database, such sample is directly uploaded to VirusTotal so that it can be incorporated and analyzed, which extends the ring execution time depending on the sample size.

All in all, the conducted experiments on the response time of the sentinel w.r.t. Android malware detection show that the time required for the malware analysis is lower the 3.1 s even in the worst case, that is, a very large file size over the slowest ring (i.e., the second one). On average, the sentinel is able to analyze the malware within a second. This allows one to easily say that the proposed solution is suitable in the context of *near real-time* detection.

Regarding the detection rate, Figure 5b depicts the detection accuracy of the three rings. In this context, we refer to detection rate as for the quantity of detected malware in a specific run. It has to be stated that, contrary to the experiments executed to measure the response time, the detection rate has been calculated enabling sequentially all the rings. In other words, the malware samples were initially analyzed by the first ring, then if Yara was not able to detect the malicious file, the second ring comes into play, and so on until the third ring. Hence, in Figure 5b, a cumulative detection rate for the malware samples is shown. A remarkable result is represented by the low detection exhibited by the first ring. That is, the static malware analysis performed with the Yara rules downloaded from the official repository [47] stuck to 8%. However, the presence of Yara in the proposed IoT detection framework relies on the fact that Yara rules can be read and compared against sample features in a fast way. Moreover, new Yara rules are being developed and contributed constantly, counting on an everyday bigger supporting community. Additionally, if a malware is able to elude the detection engine, Yara may autonomously generate an ad hoc rule which will detect the malicious trace in the future. This beneficial capability fulfills the previously described security goal of adaptability for the sentinel, as mentioned in Section 2, which we believe is of crucial importance in IoT contexts. Furthermore, adding more Yara rules implies directly a slower static analysis, which in the case of Android malware detection is not increasing concretely the detection rate.

Another interesting feature shown in Figure 5b is the absence of undetected malware. Specifically, no malware of the selected 5000 samples remains undiscovered after passing through the three rings of the sentinel. For the most part, the second ring (i.e., the machine learning module) is able to spot the malicious files, reaching a notable 75% detection rate. Lastly, the third ring (i.e., VirusTotal) is able to block all the remaining malware, summing up a 100% detection rate for COSMOS. This result can be explained looking at the operation mode of VirusTotal: when a malware hash is not present in the databases, the tool requires the upload of the entire file to process it internally. Since the Drebin dataset used during the experiments was made available in 2014, the involved malware instances have been already uploaded and analyzed by VirusTotal. Thus, for the Drebin malware, VirusTotal manages to correctly label them all efficiently and in a timely fashion.

Furthermore, Figure 5c,d depict the impact of the COSMOS architecture on the Raspberry’s resources. In particular, we registered for each experiment the CPU and RAM usage, plotting also in this case the median values. Considering the CPU consumption, Figure 5c shows that the overall CPU usage for very small Android malware (i.e., less than 100 KB) is lower than 21.5%, while for other malware sizes increases until 25% of utilization. This outcome may be explained by looking at the operations performed by the three detection modules; that is, each of them is directly influenced by the malware size, thus implying a higher computation demand. For the RAM consumption, similarly, Figure 5d reveals that the malware size straightforwardly impacts the RAM usage, since a bigger malware requires more memory to be analyzed. However, the overall RAM consumption never exceeds the 350 MB in the worst case (very large malware samples), which can be seen as acceptable in this context.

In conclusion, the experimental results on Android malware detection prove the ability of COSMOS to analyze and detect the incoming malware samples. In particular, our proposal is able to analyze most of the malware in less than two seconds, making it suitable for an online threat detection. Moreover, by combining the powerful capabilities of static rules, anomaly detection and external knowledge, the detection rate reaches 100% for the used dataset. Additionally, the impact on the resources of the Raspberry is not critical, reaching 25% of CPU usage and 350 MB of RAM usage in the worst case (i.e., very large malware samples). Thus, we can safely conclude that the proposed framework is suitable to protect IoT devices from potential Android malware-threats.

#### 4.2.2. Non-Android Malware Detection

This section describes the experiments performed to evaluate the performance of COSMOS w.r.t. the detection of generic malware. As previously mentioned, in this case, the ML is not effectively working, thus the malware samples are passed to the static detection layer (i.e., Internal Analyzer using Yara rules) and subsequently to the External Analyzer (i.e., VirusTotal).

The experiments were performed over a set of 5000 malware samples belonging to different families. The malware were originally collected from open-source databases of generic malware [48], excluding the Android samples, such as Offensive Computing [49] and Virus Sign [50], among others [51]. As previously explained, even for this experiment, the files were directly injected within the catching directory of the Sentinel, thus starting the overall analysis. In this experiment, we decided to select the following size classes: (i) less than 100 KB, (ii) from 100 KB to 500 KB, (iii) from 500 KB to 1 MB, and (iv) from 1 MB to 5 MB.

Regarding the static detection layer, we decided to increase the number of charged Yara rules in order to argue on possible differences in terms of performance. Specifically, we used 474 rules taken from the official Yara-rules repository concerning the detection of different malware families. Similarly to the Android detection testing, to discuss on the sentinel’s behavior, the following parameters were measured, as reported in Table 2: (i) the global *resource consumption* of the framework’s components (i.e., CPU and RAM consumption), (ii) *response time*, that is, the time a particular defensive layer requires giving a response about a given sample, and (iii) *detection rate*, meaning the percentage of malware samples detected for a given experiment in each layer. One could say that the above-mentioned parameters are particularly critical in the context of IoT threats’ detection.

**Figure 6:**
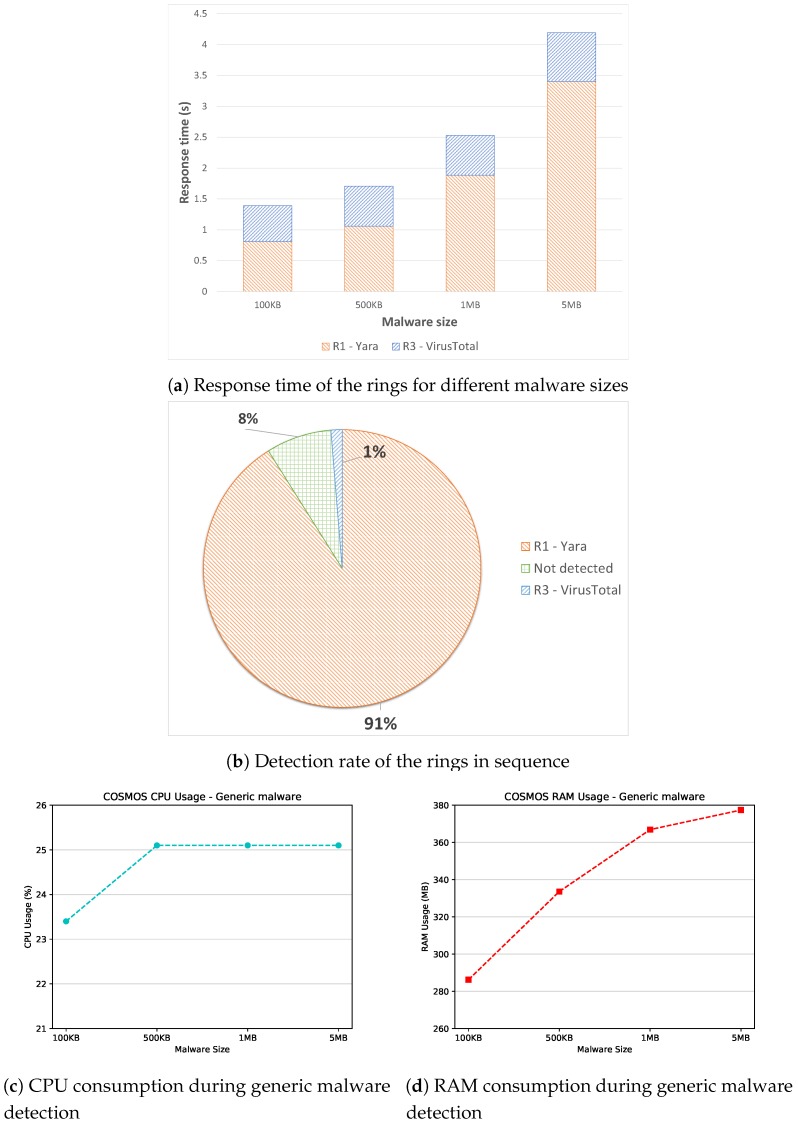
Results of the experiments conducted on COSMOS w.r.t generic malware detection.

Significant results are shown in Figure 6, where the response time of the Sentinel for different malware sizes is depicted in Figure 6a, the detection rate of the rings is showed in Figure 6b, and finally the resource consumption in terms of CPU and RAM is illustrated in Figure 6c,d respectively.

Starting with the response time, Figure 6a depicts the time required by the defensive layers to analyze the malware sample. It is worth mentioning that the response time has been calculated separately also during this experiment to accurately argue on the performance of each ring. Moreover, the median value of the obtained time values has been plotted, since it is more robust against possible outliers. Contrary to the results shown in Figure 5a, the first ring (i.e., Yara) is the slower one during the experiment. This outcome is mainly due to the number of rules used to detect the malware samples: that is, the bigger is the rule set, the slower is the overall execution, since the source code of the malware has to be checked against more rules in order to find potential matches. Thus, the time required for the static analysis to be completed increases linearly with the malware size, reaching 3.5 s for large samples. Nonetheless, the time performance of the static layer may be further improved, say, by enabling a specific subset of rules, or by studying the number of correct hits generated by a specific rule. Such improvements would decrease the overall execution time of the static ring. On the other side, as already discussed in the previous sections, the response time of VirusTotal remains quite constant, expected for large malware samples, for which the time needed to compute the hash becomes considerable.

Regarding the detection rate, Figure 6b illustrates the detection accuracy of the enabled rings. It has to be stated that also in this case we refer to detection rate as for the quantity of detected malware samples in a particular experimental run. Additionally, the presented results have been calculated enabling sequentially all the rings, differently from the experiments related to the response time. Specifically, the malware samples were initially analyzed by the static layer, then, if Yara was not able to detect the malicious file, the third ring comes into play. Hence, in Figure 6b, a cumulative detection rate for the malware samples is shown. An interesting result is represented by the high detection rate shown by Yara, which reached 91% during the experiment. This outcome is mainly explicable by looking at the higher rules number charged into the Sentinel during this experimental session. In particular, compared with the Android detection experiments presented in Section 4.2.1, we increased the number of rules from 72 to 474, which is the entire ruleset available on the official Yara-rule repository. The main disadvantage of this change, as previously mentioned, is represented by the worse time performance, while the detection is clearly improved. Thus, we can safely conclude that the Yara-rule repository is well-performing against generic malware, while the Android detection is still an on-going project.

Another interesting result shown in Figure 6b is the presence of a small portion of undetected malware, together with a low accuracy exposed by VirusTotal. This outcome is justifiable considering on the one side the higher complexity of generic malware compared with the Android ones. On the other side, the malware traces analyzed during the experiment also contain specific extensions (i.e., .bytes, .asm), which VirusTotal is not able to analyze, while the rule-based static analyzer is performing accurately with the above-mentioned extensions.

Furthermore, Figure 6c,d show the impact of COSMOS on the Raspberry’s resources w.r.t generic malware detection. Also in this experiment, we registered the CPU and RAM utilization of the detection components, plotting the median values for different malware sizes. Regarding the CPU usage, Figure 6c shows that the overall usage is around 25%, with a low peak for very small malware size (i.e., 100 KB). Comparing this outcome with the Android detection experiments, we can conclude that the different malware extensions do not impact the CPU performances, which stuck to an acceptable level. Regarding the RAM consumption, the trend is similar to the Android experiment presented in Figure 5d. That is, a bigger malware size implies directly a higher RAM usage. The main difference among the two tests is that the RAM consumption for generic malware detection is slightly higher, since more rules are charged within the static analyzer. Nevertheless, the overall RAM usage never exceeded 380 MB in the worst case, thus representing an acceptable result considering the Raspberry’s capabilities.

All in all, the experimental results w.r.t. generic malware detection demonstrate the capabilities of COSMOS in dealing with incoming malware samples. Specifically, our solution reaches the 92% of accuracy and analyzes the samples in less than 4.5 s (in the case of very large malware). Such results may be further improved by studying the Yara-rule repository, but also by developing and enabling the other protection layer (i.e., ML model), which proved its capabilities for the Android samples. Moreover, the impact on the Raspberry’s resource is not critical, reaching 25% of CPU usage and 380 MB of RAM usage in the worst case. Thus, we can conclude that the proposed architecture, also in case of generic malware detection, is suitable to protect the surrounding IoT devices from potential threats.

### 4.3. Challenges

The design and implementation of the IoT Sentinel indeed faced different challenges. The first one is related to the difficulty to reconstruct samples based on the raw data sniffed from the communication channel. This aspect was resolved by leveraging the capabilities the Ethernet (Snort) and WiFi (Kismet) monitors. However, we are also assessing whether to implement a reverse proxy over the sentinel so that the reconstruction can be more precise. A second challenge was related to the fact that Yara rules are static and require to be updated regularly. For this aspect, we are looking for public IoT-focused Yara rules databases which can feed the local Yara repository existing in the IoT Sentinel. Finally, a third challenge is about the management of data in different formats—that is, in [20], the proposed IoT Sentinel only Android applications. However, we extended the dissertation throughout this article to include other file extensions, thus demonstrating the detection capabilities of COSMOS also regarding generic malware. Nonetheless, developing a ML model which is capable of classifying different malware formats is still an open and interesting challenge.

## 5. COSMOS App

As we have seen so far, the COSMOS sentinel is able to explore its environment, finding devices in the nearby to be protected. Once any of those devices becomes a victim of an intrusion or a cyberattack, the sentinel promptly identifies such circumstance and accurately and efficiently reports an alert to a SIEM. Similarly, vulnerabilities report are sent to the SIEM platform whenever the IoT devices expose such weaknesses. Upon reception of those alerts at the SIEM, sysadmins can carry out the opportune measures to deter the attackers and restore an adequate protection level.

However, it is not realistic to assume the presence of a SIEM solution in every scenario. One good example of an environment where the sentinel can actually turn out to be extremely beneficial, even in the absence of a SIEM, would be a smart home. In here, an increasing number of devices will cohabit and even communicate with each other, the smartphone usually being a central point of control for many of those elements, principally via Apps installed on it.

In this regard, and with the aim of easing the enjoyment of the sentinel in those contexts lacking a SIEM (like smart homes), we decided to develop a friendly and easy-to-use COSMOS App (Android). It is worth mentioning that the COSMOS App is not intended to replace the SIEM platform, since it lacks some security functionalities, such as correlation rules, statistic models, IoC feeds, and so forth. Such App endows the end-users with the ability to handle and control a number of sentinels. That is to say, a given sentinel can be registered and administered by more than one App instance (for example, more than one person owning the sentinel within the same home), and one App instance can register and administer more than one sentinel (for example, for those users having a sentinel at home and another at the office).

**Figure 7:**
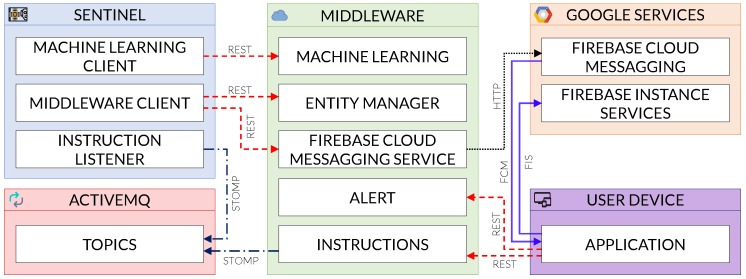
COSMOS App deployment overview.

Figure 7 depicts an overview of the deployment we employed in order to make the COSMOS App work. As it can be observed, we resorted to a central middleware acting as a services provider for both sentinel(s) and the App(s). Every alert generated by the sentinel is now transmitted to the middleware who, in turn, forwards it to all those Apps subscribed to such sentinel. Likewise, the end-users can also send commands back to the sentinel (such as countermeasures to be enforced in order to block and eradicate the attacks) using the App, via the middleware.

More specifically, the middleware provides different services divided in five areas:

- **Alert**: This area manages all the App alerts, including alert information, statistics and persistence. This area does not receive information from the sentinel, as those alerts are taken by the Firebase Cloud Messaging Service for a better treatment.
- **Entity Manager**: This area manages all the entities in the middleware, providing CRUD operations for them. An entity is the representation of an actor in the COSMOS architecture, such as a Sentinel, the App, a Device or an Alert.
- **Machine Learning (ML)**: This area manages the classification of samples into goodware or malware; it contains the ML model and, after receiving the characteristics, it returns the classification according to that model.
- **Instructions**: This area manages the exchange of instruction between the App and a selected sentinel, after receiving the instruction from the App, the middleware will publish it in the message broker, allowing for the called sentinel to read it and execute the instruction.
- **Firebase Cloud Messaging (FCM) Service**: This area receives the alert messages from the sentinel and forwards them to the App using the Google FCM API, the messages are received using the FCM instance manager, then a notification showing the message (and thus the alert) pops up.

Even with all the services provided by the middleware, there are still two more components in the architecture besides the App and the Sentinel; those components are ActiveMQ (i.e., a Message broker) and Google services. The message broker is used to communicate the middleware and the sentinel, so the asynchronous messages containing instructions are not blocking the middleware’s operation. The Google services are used in two moments of the App operation. The first one is the identification of the App provided by Google using an Instance Token; this token will not change except in extreme cases, such as reinstalling of the App or erasing its data. The second moment is the native message sending from Google FCM to the App; this is used by the middleware to deliver the alerts that the sentinels send.

**Figure 8:**
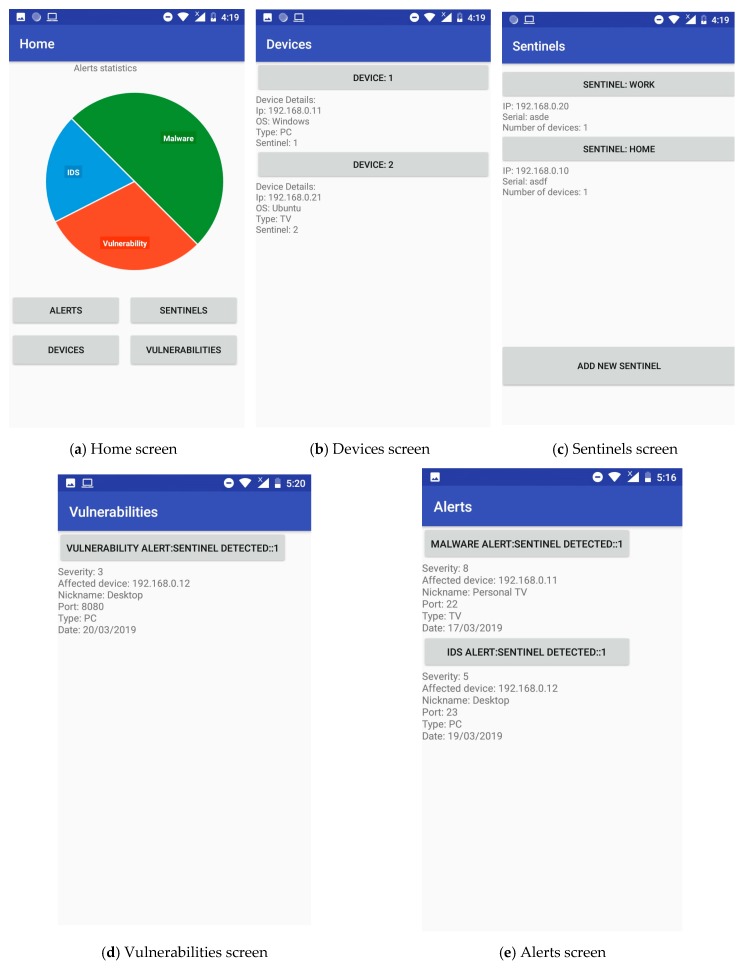
COSMOS App screenshots.

Thus, the key actions allowed by the COSMOS App are (as shown in Figure 8):

- **Home**: This screen is the entry point of the App and shows an overview of the alerts generated over the system(s) under protection of the sentinel(s) (Figure 8a).
- **Devices**: Through this screen, end-users can trigger a scanning of the environment of each of the registered sentinels in order to determine which devices should be protected (and which not). Moreover, some basic information is displayed for each signed-up device, such as operating system, IP address, vulnerabilities or pending alerts (Figure 8b).
- **Sentinels**: The binding process between sentinels and the App is performed here, allowing also to configure or even remove previously registered sentinels. Such process requires the association between the unique sentinel ID and the hash-generated App ID. As for the sentinels’ settings, things like stating a nickname (‘home’, ‘office’, etc.), for instance, are permitted (Figure 8c).
- **Vulnerabilities**: This screen provides a snapshot of the vulnerabilities found in each and every device selected to be protected by the sentinels. Some basic information regarding each vulnerability is shown, like the service or protocol affected, and the severity of the discovered vulnerability—for instance, (Figure 8d).
- **Alerts**: Last but not least, the Alerts screen gathers all the warnings submitted by the sentinels regarding intrusions and cyberattacks. For each of these alerts, further information such as the victim device, or even a number of potential countermeasures, is offered to the end-users (Figure 8e).

## 6. State of the Art

Several solutions have been presented so far in the literature aiming at addressing the security challenges raised by the IoT environments. In [52], authors proposed a secure architecture for IoT systems based on the Unit IoT and Ubiquitous (U2IoT) model. The architecture is intended to address security from three perspectives, namely, information, physical and management. Among the features shown by such proposal, intelligence is one of the most remarkable, supporting network analysis, self-adaptation to different threat scenarios and the definition of countermeasures.

Continuing with adaptations of current technologies, Ref. [53] also states that conventional security technologies and approaches are not suitable for IoT security and privacy issues. Thus, the authors’ proposal is an architecture for IoT devices that uses a lightweight version of the Blockchain (BC) technology to meet the resources’ constraints of the nodes connected to the IoT network (both internal or external ones). The architecture contains a centralized and local BC (running on the machine with most resources) that manages transactions aiming at securing both devices and connections between them. Within the architecture, the privacy is preserved with the use of the BC technology and the Tor network [54] among the IoT nodes to connect to each other. Then, the proposal is implemented in a smart home scenario, testing it against a possible set of cyber threats.

Another approach to IoT security is shown in [55], where the authors claim that the usual approach to security issues cannot be enforced to IoT ecosystem, thus proposing a systematic approach to solve this issue. The presented system consists of four nodes that interact with each other, namely, person, technological ecosystem, process and intelligent object. Each of these nodes has interactions, called *tensions*, with other nodes. Possible representations of tensions might be identification/authentication, trust, reliability, auto-immunity, privacy, responsibility and safety. Specifically, the tensions between the intelligent object and the process define the security paradigm for IoT, since the process refers to a means to accomplish tasks in the IoT environment according to its security requirements. To this extent, the authors described the essence of each tension, the effect of its application, related works and possible research issues.

Additionally, a framework based on embedded security that goes around all device cycle and conventional solutions is presented in [56]. This proposal sets a view on the embedded security for IoT devices and proposes a methodology that goes from the birth to the disposal of the device. The proposed methodology combines with an architecture that considers the hardware and software perspective to provide the embedded security without leaving unattended the environment in which the device is functioning. A proposal that protects the environment and the device is helpful, but the paper accepts that all variables may affect the architecture so, if something fails in the definition, the specifications for the framework will be wrong and security may fail.

Another security solution for IoT has been presented in [57], where authors studied and designed a framework with security embedded in each layer of the ecosystem. In particular, each layer of the proposed framework (i.e., things, communication, infrastructure and data analytics) aims to minimize the risks of some common threats for IoT, which are specifically discussed along the research. Therefore, the security paradigm is applied on each layer, so that the component devices get embedded security and data analysis through Big Data techniques. The potentialities of the entire framework are then demonstrated by developing a prototype in a cloud environment.

An alternative framework that aims at e-health and uses risk management is proposed in [58]. Such framework estimates and predicts damages by doing risk management and context awareness techniques, as well as using game theory in the prediction model. The behaviour prediction of a lot of stakeholders and interested actors in the IoT is fascinating, and, in cases like a smart home or places with more computing capability, it could be more useful.

Furthermore, a traffic-aware patching scheme to face malware attacks in IoT scenarios is introduced in [59]. This proposal claims to be more practical and efficient to patch intermediate nodes (e.g., access points, base stations or gateways), which have wired connectivity for data relay, to avoid that an attack spreads, and thus limiting the malware propagation to device–device connections. Nodes can be selected via a novel “traffic-ware” patching algorithm based on traffic volume (the ones with the highest metric being firstly patched) and actually patched using *Over The Air* (OTA) update mechanisms.

Another research work is presented in [60], where a wireless intrusion detection approach is proposed. Specifically, the authors developed a neural network algorithm to characterize legitimate communications’ profiles, and thus they are able to distinguish them from suspicious behaviors at the physical layer. A profile of the Radio Signal Strength Indication (RSSI) is calculated in strategic points of the proposed scenario (i.e., smart homes or smart factories) and used as a baseline to define legitimate areas where devices usually communicate. Then, preliminary experiments are performed to validate the assumptions, showing promising results.

Similarly, authors in [61] present a novel IDS based on previous threat analysis. This solution leverages the capabilities of an Artificial Neural Network (ANN). Particularly, the ANN used in this context is a multi-level perceptron (i.e., a supervised ANN), which was trained to detect DDoS attacks in the network. The detection is based on the classification of the network data into normal or threat pattern, relying on data learned by the ANN.

In the same line than the previous proposal, Ref. [62] proposes a solution that detects unauthorized devices within the IoT network by using Machine Learning (ML) techniques (i.e., Random Forest classifier [63]) to determine the actual nature of the devices. First, the classifier was trained with manually labeled instances, and then the detection is done by monitoring the real network traffic. The devices are classified using a white list of authorized device types. The algorithm is able to classify the new device either as one of the authorized ones or as unknown and unauthorized.

Likewise, Ref. [64] shows the results of using an IoT honeypot (IoTPOT) and a sandbox (IoTBOX) to analyze Telnet attacks and malware samples, respectively. The IoTPOT is composed of a module emulating IoT devices and managing: connections, banner interactions, authentication and command interactions. From this research, it was concluded that there are five malware families exploiting telnet-enabled devices, which are focused mainly in DDoS attacks.

In addition, the proposal in [65] is to monitor the network and to use Software Defined Networks (SDN) to block or quarantine the devices based on detected suspicious behaviours. To this extent, an SDN-enabled architecture is defined to describe the elements and the interactions among them. Specifically, the architecture is divided in three parts: (i) the Security Management Provider (SMP, which provides protection mechanisms at the network level for the IoT devices), (ii) Home route vendor (i.e., gateway that could allow SMP to configure network behavior) and (iii) the consumer (i.e., user in the household). Lastly, a prototype of the overall architecture is then developed using an open-source SDN platform, showing the actual feasibility of the proposal.

Another approach is proposed in [18], where an architecture of IDSs for IoT ecosystems is presented. The novel architecture utilizes single-board computers that are pre-packed with an IDS and are also portable and small, following the premise of Plug and Protect. The validation is performed employing a Snort IDS running over a Raspberry Pi 2 Model B unit and measuring variables like CPU and RAM usage, Packet Capture rate and number of generated alerts. Similarly, authors in [3] extended such IDS architecture by adding the wireless detection capability, considered of vital importance in the IoT scenario, and integrating it with a SIEM. The authors claim that the presence of a remote SIEM server capable of collecting data stemming from different sources represents a primary need. Using a SIEM platform, the incoming events are filtered and correlated, lowering the false positives and increasing the situational awareness.

One more type of response to IoT threats is described in [16], where a security sentinel is designed using a *Brownfield approach* to protect legacy and new IoT devices. The sentinel identifies the type of connected devices, and therefore its potential security issues, by capturing fingerprints that fed a two-fold classification system. The sentinel also enforces security through the definition of isolation levels (strict, restricted and trusted) and through mitigation strategies (network isolation, traffic filtering and user notification).

All in all, different approaches have been proposed in the literature aiming to improve the security around the IoT ecosystem. The reviewed works addressed this cumbersome topic by presenting diverse solutions, from ad hoc network protocols to innovative architectural schemas, as summarized in Table 3. Specifically, Table 3 shows that, although these proposals exhibit good features, they mostly lack two highly-desired security goals, namely adaptability (i.e., learning from the current threat context) and collaboration (i.e., sharing information with other sentinels). Within the IoT context, characterized by high nodes’ mobility and dynamism, one could argue that these objectives are fundamental to ensure an adequate level of protection to the devices. As described in the previous sections, the work at hand proposed a novel sentinel that, besides being automatic, non-invasive and easy to deploy, it is also collaborative, reactive and adaptive, thus filling the gap exposed by the literature.

## 7. Conclusions and Future Work

The IoT paradigm is progressively integrating within our everyday life. Consequently, smart objects are already located in several daily-used appliances, exchanging massive amounts of information among them. The great benefit carried by the IoT world does not come without a cost: several security issues are raised around it, as it is stated in the literature, where worldwide researchers are struggling to propose innovative solutions to address the posed challenges.

In this paper, we presented COSMOS, a novel framework to protect IoT environments against cyber-threats. Our sentinel is able to perform its detection duties using multiple protection rings, which combine the capabilities of the rule-based and anomaly-based detection to improve the detection accuracy as an ultimate goal. COSMOS is able to operate seamlessly and autonomously, alerting the final users in case of discovered intrusions. Furthermore, it is able to adapt to previously unknown threats and to share the gathered knowledge with other sentinels. To demonstrate the feasibility of COSMOS, exhaustive experiments have been conducted. During the above-mentioned tests, the sentinel has been heavily stressed with several malicious frames, reproducing a wireless attack scenario. Then, it has been intensively tested with diverse malware instances of different sizes and families. Experimental results demonstrate that the proposed sentinel is able to perform an accurate and fast detection across the different protection layers. Furthermore, with the aim of easing the enjoyment of COSMOS in other scenario, such as smart homes, we developed a friendly Android App to remotely control the sentinels and protect the home devices.

Future works will explore the possibility of leveraging the SIEM system to collect events stemming from different sentinels and to automatically react to intrusions. Moreover, a scenario in which the architecture is employed to detect distributed attacks, such as DDoS, is worthy of investigation. Moreover, we are currently studying the development of a ML layer which can automatically recognize and classify different malware extensions. Further experiments considering different throughputs (mbps) in the communication channel are also expected to be performed so to test the ability of the sentinel to intercept samples in a real-case scenario.

## Figures and Tables

**Table 1:** Monitored statistics and input parameters for COSMOS intrusion detection experiments.

Category	Name	Description
*Statistics*	CPU	Raspberry Pi CPU usage along an experiment time lapse
	RAM	Raspberry Pi RAM usage along an experiment time lapse
	Analyzed packets	No. of packets analyzed from the IDSs
*Input*	RuleSets	{connectivity, balanced, security}
	Detection Algorithms	{lowmem, ac-bnfa, ac-split}
	Time window	8-h

**Table 2:** Monitored statistics during the experiments.

Name	Description
CPU	Raspberry Pi CPU usage for a given experiment
RAM	Raspberry Pi RAM usage for a given experiment
Response time	Time required to analyze a sample
Detection rate	Percentage of detected malware

**Table 3:** Comparative table of the analyzed related works.

Related Work	Methodology	Scenario	Security Goals			
			*SG*_1_	*SG*_2_	*SG*_3_	*SG*_4_
Ning and Liu [52]	Security from three perspectives	IoT ecosystem	N.A.	**✗**	**✓**	N.A.
Dorri et al. [53]	Adapt current technology to IoT Scenario	Smart Home	**✓**	N.A.	**✗**	**✓**
Riahi et al. [55]	Systematic approach to improve IoT security	IoT ecosystem	N.A.	N.A.	N.A.	N.A.
Babar et al. [56]	Embedded security in all stages of device lifecycle	IoT ecosystem	**✓**	**✗**	**✗**	**✓**
Rahman et al. [57]	Embedded security in each layer of the IoT ecosystem	IoT ecosystem	**✓**	**✗**	**✓**	**✓**
Abie and Balasingham [58]	Risk management based security	e-Health	N.A.	**✗**	**✓**	N.A.
Cheng et al. [59]	Traffic aware patching intermediate nodes	IoT ecosystem	**✓**	**✗**	**✗**	N.A.
Roux et al. [60]	Identification of suspicious behavior at physical layer	IoT network	**✓**	**✗**	**✗**	**✗**
Hodo et al. [61]	Detection of DDoS attacks	IoT network	**✓**	**✗**	**✓**	**✗**
Meidan et al. [62]	Detection of unauthorized devices in the network	IoT network	**✓**	**✗**	**✓**	**✗**
Pa et al. [64]	Honeypot and Sandboxing	IoT ecosystem	**✗**	**✗**	**✗**	**✓**
Sivaraman et al. [65]	SDN Networks to detect and block devices	IoT network	**✓**	**✗**	**✗**	**✗**
Sforzin et al. [18]	Single-board computer IDS	Smart Home	**✓**	**✗**	**✗**	**✓**
Nespoli and Gómez Mármol [3]	Wireless IDS with SIEM integration	e-Health	**✓**	**✗**	**✓**	**✓**
Miettinen et al. [16]	IoT Sentinel to protect and identify IoT nodes	IoT network	**✓**	**✗**	**✗**	**✗**
